# War and Population Health: there is no metric for the horror

**DOI:** 10.1186/s12963-023-00321-x

**Published:** 2024-01-30

**Authors:** Jonathan M. Samet

**Affiliations:** Colorado School of Public Health, Aurora, CO USA

I write this commentary as December 2023 ends, a year marked by continued war between Russia and the violated country of Ukraine, and by the explosive conflict between Israel and Hamas that began with the Hamas’ attack on Israel on October 7. Some statistics from these conflicts can be readily found with a Google search, albeit with disclaimers as to their accuracy. For the Ukraine conflict, an August 2023 report from US intelligence estimated a total of almost 500,000 killed or wounded military personnel—about 300,000 Russians and almost 200,000 Ukrainians [1]. The United Nations estimates 10,000 casualties among Ukraine’s civilians, including more than 560 children [2]. The figures at the moment for the Israel/Hamas conflict are at least 18,787 killed in Gaza, including 7700 children, and about 1147 were killed in the October 7 Hamas attack [3]. Likely undercounts, these numbers do not capture the indirect burden of mortality posed by conflict nor the suffering and future morbidity from these conflicts.

Moving beyond counts of the dead, Barry S. Levy describes the full scope of the impact of war on health in “From Horror to Hope. Recognizing and Preventing the Health Impacts of War” [4]. This 2022 book is systematic in its coverage of how war affects health. The enormity of what Levy describes is overwhelming, and we now see it happening in real time. When Russia invaded Ukraine, I could not stop watching the initial days of conflict as the seemingly unimaginable happened. Levy’s approach is systematic, beginning with coverage of war itself and then moving on to weaponry, health impacts on civilians, and other issues, including the health impacts on military personnel and the environment and the assessment of the health consequences of war. The last chapter addresses the need to do what has been impossible—to prevent war, the root cause in the series of war-initiated causal chains that harm human health and the environment.

The chapters are interrupted by hopeful vignettes of people who are making a difference in contending with war and its health consequences. These depictions offer reminders that actions by committed individuals can have impact, whether through healthcare delivery or policy actions. These individuals are the heroes of “From Horror to Hope.”

Readers of *Population Health Metrics* will be most interested in Chapter 14: “Determining the Health Impacts of War.” The chapter starts with seven major challenges to doing so with accuracy: (1) lack of security and stability; (2) inaccurate reporting; (3) inadequate data systems; (4) displaced populations; (5) indirect health impacts; (6) distant impacts; and (7) delayed impacts. The current situation in Gaza exemplifies the first four challenges: The on-the-ground situation is prohibitively dangerous, data systems are lacking, numerous powerful forces may bias mortality counts and morbidity figures, and most of the population has been displaced. The last three—indirect health impacts and distant and delayed impacts—are inevitable, but any projections made now for Gaza and Israel would be highly uncertain.

Nonetheless, estimates have been made of the human toll of war. Levy catalogues the numbers of civilian deaths in six wars, e.g., 2.1 million Vietnamese civilians in the Vietnam war and civilian deaths and also injuries from landmines and unexploded ordinance. In the latter category, children are often the victims. Chapter 14 reviews selected epidemiological studies carried out in challenging circumstances, e.g., the Syrian Civil War and the US invasion of Iraq. Given the implications of such estimates, they will be closely scrutinized and may be politicized. For example, Roberts et al. estimated a toll of more than 100,000 excess deaths among Iraqis during the 18 months following the invasion of the country by the USA [5]. A follow-up report covering 40 months post-invasion escalated that estimate to more than 650,000, a figure contested by then President George Bush [6, 7].

Reports of the numbers of casualties and injured, particularly among civilians, may help to motivate an end to a conflict and also lead to calls to protect civilians, as with the rapidly increasing numbers of civilian deaths in Gaza at the moment. Even with the challenges described by Levy in play, the high death toll of civilians in Gaza, particularly children, has led to vigorous calls for a change in Israel’s tactics. More certain estimates from validated methods might have greater influence.

What has *Population Health Metrics* contributed with regard to methods for addressing the burden of morbidity and mortality from war? A search of all publications in the journal using the terms war and conflict identified a few relevant articles. One of the most directly relevant is the 2022 paper by Chechhi and colleagues, who describe a small area estimation method and illustrate its application in Somalia, South Sudan and northeast Nigeria [8]. A few studies address issues related to the health of military veterans.

Given Levy’s seven challenges and the paucity of relevant methodological papers in *Population Health Metrics*, I encourage submissions on assessing the impact of war on population health. “From Horror to Hope. Recognizing and Preventing the Health Impacts of War” will provide needed background. Paraphrasing Levy’s title, better evidence on the horror of war may bring more hope.
